# Doctors and Socialism

**Published:** 1908-07-11

**Authors:** 


### Doctors and Socialism.

When Adeimantus put his inconvenient question as to what was to become of the individual happiness of the guardians of the ideal republic, if they were to be denied personal property and other pleasant things, he was at once silenced by the reply that their only *raison d'être* was the good of the State. The military profession is not the only one to which this generous principle—"Work of each for weal of all"—has been recommended. There are plenty of quite modern instances where our own relations with the rest of the body politic have been examined from the Collectivist point of view. In his last book, Mr. H. G. Wells again touched on that rather favourite topic of his—namely, that under present conditions the medical man is a parasite of disease. Lately Mr. Lawson Dodd preached the nationalisation of the profession to a Manchester audience. More recently still, Dr. Eder has written to the same effect in the columns of a lay contemporary, the well-advertised brilliance of whose occasional contributors has won for it a distinct success. It is comforting, however, to find that the Fabian doctrine is not so stern and uncompromising as the Socratic one. It condescends to point out (with some justice) that our present lot is hardly of the happiest; and to predict that so long as the vast bulk of us live by fees derived from sick persons, so long shall we continue to be defrauded of much of our just remuneration by patent medicine vendors and empirics, and perhaps driven to ignoble expedients, which last, it may be added, were strikingly exemplified by a recent advertisement in a well-known medical journal, where actually Dalmatian dogs were quoted for sale, and described as "suitable to run behind a doctor's carriage." The critical part of the Socialist propaganda is always easy. For instance, the process by which our clinical experts are produced is undoubtedly a wasteful one. Scores of capable consultants sit idle in highly rented chambers in the Cavendish Square quarter for the most productive years of their life, and only obtain at all a vogue when but a comparatively few years of practice remain to them. Nobody seems to want their services at a moderate figure, and to engage wholeheartedly in research would extinguish their chance of hundred guinea fees and an ultimate competence.

The constructive problem, of course, is to reconcile repression of undue competitive individualism with maintenance of a high standard of production. The first-class clinician, particularly the first-class surgeon, seems often of the type of successful barristers or politicians, men who love struggle and aggression, and to whom wealth and material advantages appeal rather strongly. If these incentives were removed, could they be got to practise themselves in their art with the same assiduity? Mr. Wells has instanced the self-sacrificing and gratuitous zeal of the medical staff of our great hospitals. This is admitted, but then "back of" it all, as Americans say, is in a sense the honorary's actual private practice or the resident's prospective one. If Plato tacitly acknowledged his vision of social solidarity to be impracticable, and if in all the centuries since it has never been realised, present prospects do not seem very hopeful. The scientific method of inquiry, however, is comparatively a new thing, and through it a solution may be found.

